# Linear and nonlinear optical absorption coefficients in InGaN/GaN quantum wells: Interplay between intense laser field and higher-order anharmonic potentials

**DOI:** 10.1016/j.heliyon.2023.e22867

**Published:** 2023-11-25

**Authors:** Redouane En-nadir, Mohamed A. Basyooni-M. Kabatas, Mohammed Tihtih, Haddou El Ghazi

**Affiliations:** a University of Sidi Mohamed Ben Abdullah, Fez, B.P. 2202, Morocco; b Dynamics of Micro and Nano Systems, Department of Precision and Microsystems Engineering, Delft University of Technology, Mekelweg 2, 2628, CD, Delft, Netherlands; c Department of Nanotechnology and Advanced Materials, Graduate School of Applied and Natural Science, Selçuk University, Konya, 42030, Turkey; d Space Research Laboratory, Solar and Space Research Department, National Research Institute of Astronomy and Geophysics, Cairo, Egypt; e Institute of Ceramic and Polymer Engineering, University of Miskolc, Miskolc, 3515, Hungary; f 2SMPI Group, ENSAM, University Hassan-II University, Casablanca, 20670, Morocco

**Keywords:** III-Nitrides, Nanostructure, Absorption, ILF, Harmonicity

## Abstract

This computational investigation delves into the electronic and optical attributes of InGaN/GaN nanostructures subjected to both harmonic and anharmonic confinement potentials, coupled with the influence of a nonresonant intense laser field (ILF). The theoretical framework incorporates higher-order anharmonic terms, specifically quartic and sextic terms. The solutions to the Schrödinger equation have been computed employing the finite element method and the effective mass theory. Moreover, linear and third-order nonlinear optical absorption coefficients are derived via a density matrix expansion. Our analysis reveals the feasibility of manipulating electronic and optical properties by adjusting confinement potential parameters, system attributes, and laser field intensity. In addition, the ILF induces remarkable modifications, characterized by reduced resonance peak amplitudes and a blue shift in absorption coefficients. Intriguingly, regardless of potential harmonicity, the impact of incident electromagnetic intensity is notably more pronounced in the absence of the ILF. These findings hold significant promise for advancing theoretical predictions, providing valuable insights into the intricate interplay between confinement potentials, laser fields, and their effects on electronic and optical behaviors within nanostructures.

## 1 Introduction

Recent advances in the nonlinear optical properties of semiconductor low dimensional systems have shown great promise for a wide range of applications, including optical communication, biomedical imaging and therapy, biophotonic sensing, microfabrication, and optical limiting [[1], [2], [3], [4], [5]]. One of these promising nanostructures is InGaN-based quantum structures, which are a blend of Indium, Gallium, and Nitride, and are pivotal in modern applications. These materials possess tunable properties due to the controlled inclusion of indium. Their optical and electronic attributes make them crucial for devices such as LEDs, lasers, and photodetectors, offering high efficiency and superior light emission. Beyond lighting, InGaN quantum structures also play a significant role in emerging technologies like quantum information processing [[4], [5], [6], [7]]. InGaN/GaN heterostructures have been intensively investigated during the last few years due to their interesting and unique properties, such as high electron mobility, wide bandgap, strong emission in the visible spectrum, and strong polarization effects, making them highly attractive for a wide range of applications in optoelectronics, power electronics, and solid-state lighting [5,[8], [9], [10], [11], [12], [13]]. Cubic group-III nitrides are promising arsenic-free contenders for next-gen electronic and optoelectronic applications, but realizing a complete composition range has been challenging due to a miscibility gap. Researchers have been to overcome this challenge by fabricating cubic InGaN films on template substrates using PAMBE and closing the miscibility gap. X-ray diffraction and scanning transmission electron microscopy reveal the composition, phase purity, strain, and CuPt-type ordering of the films. The emission energies range from 3.24 to 0.69 eV with a quadratic bowing parameter of 2.4 eV [14].

Their properties can be tuned by the potential energy behavior. However, the potential of a harmonic oscillator (HO) is a widely used model in quantum mechanics to depict the vibrations of a molecule in the immediate vicinity of a stable equilibrium point. Additionally, it is one of the few particular quantum systems that have exact, analytical solutions. Incorporating anharmonic oscillator potentials (AHO) into the HO potential is to certainly improve the description of molecular vibrations within semiconductor nanostructures. The AHO potential characterizes an oscillator that deviates from simple harmonic motion due to the non-linear relationship between the restoring force and displacement. By incorporating higher-order anharmonic terms, like cubic, quartic, and sextic terms, into the potential, the accuracy of the harmonic oscillator (HO) approximation is enhanced, especially for large displacements from the equilibrium position. Studies on the influence of intense laser field (ILF) on the optical properties of the HOs and single and double AHOs concerned, among others, quantum tunneling [15], which could provide a deeper understanding of quantum tunneling time. It can also be beneficial for molecular spectroscopy [16] and nonlinear optics, providing information on the nonlinear optical properties of these oscillators, with potential applications in optical communications [17] and laser technology [18]. Moreover, it is relevant to diverse fields of materials science, where it could help in the deep understanding of the electronic and optical properties of materials. This may find potential applications in various areas such as optoelectronics [19], photonics [20], solar cell research [21], as well as quantum control, which may have potential applications in quantum computing and quantum information [[8], [9], [10]]. Research on anharmonic carbon nanotubes (CNTs) explores deviations from ideal harmonic behavior, leading to complex mechanical, thermal, and optical properties. Recent studies focus on thermal conductivity, phonon transport, and mechanical responses, offering insights for nanoelectronics and energy storage applications. Anharmonic CNTs hold promise for tailoring properties, advancing nanotechnology [[25], [26], [27]].

In 2013, Landi and Oliveira demonstrated that heat can be transported along a chain of particles that interact through a combination of quartic and harmonic potentials and are connected to heat reservoirs at each end [28]. They found that adding energy-conserving noise to the system causes the heat to obey Fourier's law. In turn, Fletcher's theoretical work [29] concludes that the term “anharmonic” is appropriate for describing classical oscillators, like metal bars, that exhibit non-harmonic vibrational spectra, even at small vibrations. Both the repulsive harmonic oscillator and its extended version, which includes an additional linear anharmonicity term, have been subject to examination using both exact and perturbative techniques [[30], [31], [32]]. Panek and coworkers have conducted [33] a theoretical investigation on vibrational configuration interaction, exploring both harmonic and anharmonic coupling. They employed the matrix diagonalization technique to compare the perturbative solution, computed up to the second order, with the exact solution for systems in coherent and Schrödinger cat states. They also proposed an algorithm that can be used to localize normal modes in specific subsets. This method limits errors caused by harmonic couplings and maximizes the localization of the normal modes. In their interesting work, they embedded an interaction potential with quadratic and quartic terms within an infinite square well potential of the appropriate width. They used its sine eigenfunctions as the basis functions for their diagonalization matrix method in their work.

Unlike the harmonic oscillator, the anharmonic oscillator is a truly delicate problem that cannot be solved analytically. As a result, both perturbative and nonperturbative effects have been thoroughly examined [[34], [35], [36], [37], [38], [39]]. The anharmonic oscillator potentials with sextic, octic, and other terms have also been extensively studied [[25], [26], [27], [28], [29]]. This work demonstrates the convergence of the perturbation series and explains why the standard perturbation theory is not effective for anharmonic oscillators. It also suggests a correct limit for both large and small anharmonicities. Furthermore, this study investigates the electronic and optical properties of the HO and single and double AHOs, including higher-order anharmonic terms such as quartic and sextic, under the influence of non-resonant intense laser fields (ILF). ILF plays a pivotal role in scientific research by facilitating precise control and manipulation of physical and chemical processes, with profound implications in fields such as ultrafast spectroscopy, materials science, quantum technology, atomic and molecular physics, plasma physics, medical and biological applications, materials processing, and advanced imaging. Numerous studies have extensively documented the effects of intense laser fields (ILF) on optical and electronic properties in the scientific literature [[45], [46], [47], [48], [49], [50], [51], [52], [57], [58], [59], [60], [61], [62], [63]].

Our study introduces novel aspects of confinement potential, incorporating both harmonic and anharmonic terms, including up to the sextic power. This innovation accurately represents particle behavior in quantum heterostructures, with anharmonic potentials capturing essential physical properties like energy spectra, nonlinearity effects, quantum tunneling, and temperature dependencies. Integration of higher-order terms (quartic and sextic) enhances precision in describing energy levels and eigenstates. Furthermore, our research leverages the pivotal role of intense laser fields, enabling precise control of physical and chemical processes. It has far-reaching implications in fields like ultrafast spectroscopy, materials science, quantum technology, atomic and molecular physics, plasma physics, medical and biological applications, materials processing, and advanced imaging. The extensive documentation of intense laser fields' effects on optical and electronic properties in scientific literature underpins our study. Our unique combination of these elements offers a comprehensive exploration of confined particle dynamics within quantum heterostructures under the influence of intense laser fields, with a continued commitment to advancing precision in our research.

## 2 Theory

In this study, we consider a $\text{GaN}/{\text{In}}_{0.1}{\text{Ga}}_{0.9}N/\text{GaN}$ nanostructure (quantum well), where the well is made out of InGaN, while GaN represents the barrier semiconductor material. In the effective mass approximation, the Hamiltonian for a confined electron in the investigated system is given by

(1) $$H=-\frac{\hbar^{2}}{2}\vec{\nabla }\left(\frac{1}{m_{e}^{*}}\right)\vec{\nabla }+V_{c}\left(z\right)$$

The above equation has been solved within the framework of the effective mass and parabolic band approximation. The effective mass modifies the electron's behavior in crystalline materials, simplifying equations for studying semiconductors and charge transport. Parabolic band approximation simplifies energy bands near the extrema, aiding the analysis of electronic behavior in materials.

In the above equation, the electron's effective mass is denoted by $m^{*}$, while $V_{c}\left(z\right)$ represents the confinement potential that includes harmonic and anharmonic potentials along the growth direction (*Z*-axis) for different terms (double, quartic, and sextic). The functional form of this confinement potential before the application of ILF is as follows [53]:

(2) $$V_{c}\left(z\right)=V_{0}\left[\beta_{1}{\left(\frac{z}{k}\right)}^{2}+\beta_{2}{\left(\frac{z}{k}\right)}^{4}+\beta_{3}{\left(\frac{z}{k}\right)}^{6}\right],$$

$\beta_{1},\beta_{2},\beta_{2}$and k are dimensionless parameters while $V_{0}$ denotes the depth of the quantum well, k is a parameter related to its width, and the coupling parameter $\beta_{1}$, which also affects the well width, is known as the coupling parameter. As $\beta_{1}$ increases, the well becomes narrower. The confinement potential can take the form of a single or double AHO potential, depending on the values of the parameters $\beta_{2}$ and $\beta_{3}$.

In this study, we aim to investigate the effect of intense laser fields on the optical properties of InGaN/GaN heterostructures regardless of the size of both the well and barriers. Therefore, we have fixed the design of our system with arbitrary lengths and adjusted the values of $\beta_{1},\beta_{2},\beta_{2}$ and k to shift from a Harmonic to an Anharmonic potential. So that, when $\beta_{1}\neq 0$ and $\beta_{2}=\beta_{3}=0$, the potential is called HO potential. If, however $\beta_{1}\neq 0$ (with a negative value), $\beta_{3}\neq 0$ and $\beta_{2}=0$, the potential becomes a simple sextic AHO potential. Besides, if $\beta_{1}\neq 0$, $\beta_{2}\neq 0$ (with a positive value), and $\beta_{3}=0$, the potential becomes a simple quartic AHO potential.

The laser radiation, which is non-resonant and monochromatic, is aligned parallel to the growth direction (Z) with a frequency *Ω*. Based on the method proposed in Ref. [54], when intense THz laser radiation is applied, the potential described in Eq. (2) takes the form known as “dressed-laser” potential, given as follows [[32], [33], [34]]:

(3) $$\left\langle V\left(z,\alpha_{0}\right)\right\rangle =\frac{\Omega }{2\pi }\int_{0}^{\frac{2\pi }{\Omega }}V\left(z+\alpha_{0}\;\sin\left(\Omega t\right)\right)dt,$$

where the laser-dressing intensity parameter, $\alpha_{0}$, is defined as $eF_{0}/m^{*}\Omega$, with $F_{0}$ denoting the strength of the laser field and *e* - the electron charge. Additional information on the equations presented for dressed potentials and the non-perturbative method appeals to Kramers–Henneberger translation transformation for describing atomic behavior under intense high-frequency ILF.

In optical physics, three distinct terms describe different aspects of optical absorption. The Linear Optical Absorption Coefficient (LOAC) measures how a material absorbs light linearly concerning its intensity, providing information about its transparency and absorption characteristics under low-intensity illumination. On the other hand, the Non-linear Optical Absorption Coefficient (NLOAC) considers the non-linear absorption of light at higher intensities, where the response is more complex due to multiple photons interacting with the material simultaneously and it depends on the incident electromagnetic intensity. NLOAC accounts for the non-linear behavior of materials under intense illumination. Lastly, the Total Optical Absorption Coefficient (TOAC) combines both the linear and non-linear absorption processes, providing a comprehensive measurement of a material's total absorption behavior across different light intensities. After obtaining the energies and corresponding wave functions, the linear, nonlinear, and total optical absorption coefficients for transitions between any two electronic states are calculated using perturbation expansion and density matrix methods [[36], [37], [38]]:

(4) $$\alpha^{\left(1\right)}\left(\omega \right)=\hbar \omega \sqrt{\frac{\mu }{\varepsilon_{r}^{*}\varepsilon_{0}}}\frac{\Gamma_{if}|M_{fi}|²\sigma_{\nu }}{{\left(E_{fi}-\hbar \omega \right)}^{2}+{\left(\hbar \Gamma_{if}\right)}^{2}}\text{,}$$

(5) $$\alpha^{\left(3\right)}\left(\omega ,I\right)=-\sqrt{\frac{\mu }{\varepsilon_{r}^{*}}}\left(\frac{I}{2n_{r}^{*}\varepsilon_{0}c}\right)\frac{{4\Gamma }_{if}{\left|M_{fi}\right|}^{4}\sigma_{\nu }\hbar \omega }{{\left[{\left(E_{fi}-\hbar \omega \right)}^{2}+{\left(\hbar \Gamma_{if}\right)}^{2}\right]}^{2}}\times \left[1-\frac{{\left|M_{ff}-M_{ii}\right|}^{2}}{{|2M}_{ff}{|}^{2}}\frac{{\left(E_{fi}-h\omega \right)}^{2}-{\left(\hbar \Gamma_{if}\right)}^{2}+2E_{fi}\left(E_{fi}-h\omega \right)}{E_{fi}^{2}-{\left(\hbar \Gamma_{if}\right)}^{2}}\right]\text{,}$$

(6) $$\alpha^{\left(TOAC\right)}\left(\omega ,I\right)=\alpha^{\left(1\right)}\left(\omega \right)+\alpha^{\left(3\right)}\left(\omega ,I\right)\text{,}$$

In the above equations, $\varepsilon_{r}^{*}=n_{r}^{*2}\varepsilon_{0}$ represents the real part of permittivity, $\sigma_{\nu }$ is the carrier density, $\Delta E_{fi}$ denotes the energy difference between two different electronic states (f-final, i-initial), while $M_{fi}$ is the dipole matrix element between allowed eigenvalues for incident radiation polarized in the Z− axis. Besides, $\Gamma_{fi}$ denotes the relaxation rate, c is the speed of light in free space, and I represents the intensity of the incident photon beam that leads to intersubband optical transitions. It should be noted that $M_{ff}$ and $M_{ii}$ are equal to zero due to the even symmetry of the confinement potentials.

## 3 Results and discussion

The numerical calculations have been carried out using the following parameters appropriate for $\text{GaN}/{\text{In}}_{0.1}{\text{Ga}}_{0.9}N$ nanostructures, proposed in our previous works [[38], [39], [40]]. For GaN (InN): $m_{GaN,InN}^{*}=0.20m_{0}\left(0.11m_{0}\right)$ (where $m_{0}$ is the free electron mass), $\varepsilon_{r}^{*GaN,InN}=9.6\varepsilon_{0}\;\left(10.5\varepsilon_{0}\right)\text{,}$ and $Eg^{GaN,InN}=3,410\;\left(720\right)\;meV$. For $l=2L=2a^{*}$ and x=0.1(10%): $E_{g}^{InGaN}=2,797meV,\Delta E_{g}=613.4meV$ (energy difference between barrier and well materials) and $V_{0}=429.1\;meV$. Besides, $\sigma_{\nu }=2\times 10^{24}m^{-3},n_{r}^{*}=\sqrt{\varepsilon_{r}^{*}/\varepsilon_{0}},\Gamma_{12}=1∕\tau_{12}={\left(0.5\;\text{ps}\right)}^{-1}$ and $I=0.4\;\text{MW}/cm^{2}\text{.}$ In our calculations, we use effective units, where the effective Rydberg is adopted as the unit of energy, and the effective Bohr radius is taken as the unit of length. The effective Bohr radius and Rydberg are defined as follows: $a_{b}^{*}=\frac{4\pi \varepsilon_{b}^{*}\hbar^{2}}{m_{b}^{*}e^{2}}\approx 2.55\;\text{nm}$ and $R_{b}^{*}=\frac{m_{b}^{*}e^{3}}{2(4\pi \varepsilon_{b}^{*}{\hbar )}^{2}}\approx 0.0291\;\text{eV}$, where $\varepsilon_{b}^{*}$ and $m_{b}^{*}$ are the relative permittivity and electron effective-mass of the barrier semiconductor (GaN).

**Fig. 1:**
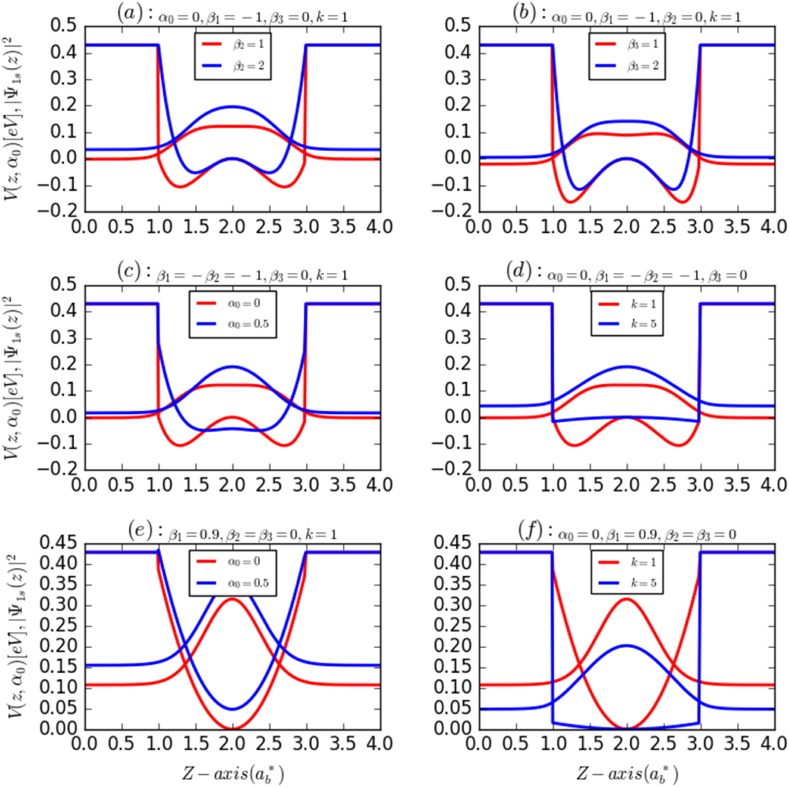
Confinement potential profile and the ground state probability density of an electron confined in both AHO (a,b,c,d) and HO (e,f) potential as a function of the growth direction parameters: the effects of the “dressed-laser” intensity ($\alpha_{0}$) and $\beta_{1},\beta_{2},\beta_{3}$ and k parameters.

**Fig. 2:**
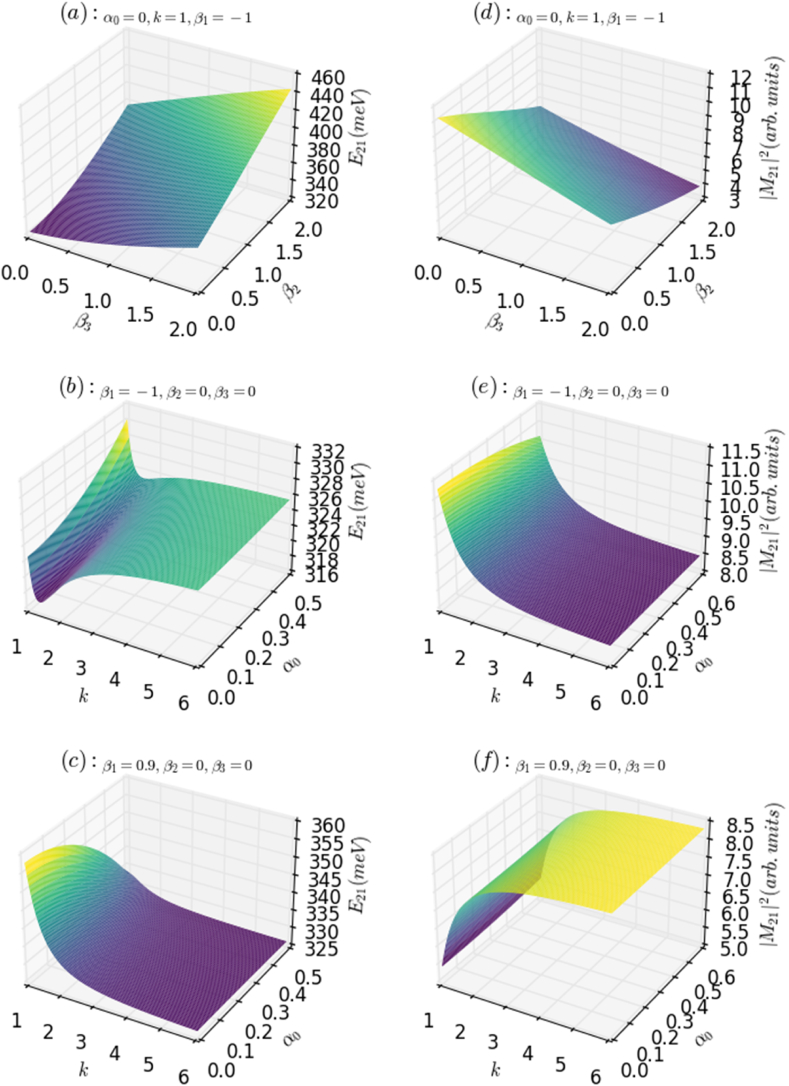
Variation of the electron transition energy [left panels (a, b, c)] and the square of dipole matrix element [right panels (d, e, f)] as a function of ${\alpha_{0},\beta }_{1},\beta_{2},\beta_{3}\text{,}$ and k..

These parameters together with the laser intensity are then used to study their effects on the LOAC, NLOAC, and TOAC. In the absence of the ILF ($\alpha_{0}=0$), Fig. 1a and 1b show the AHO potential and the squared wave function (electron probability distribution) for the first energy level with the most relevant energy value (ground state energy) for k=1 and $\beta_{1}=-1$ as a function of Z-direction (growth direction) coordinates for $\beta_{2}\neq 0$ and $\beta_{3}\neq 0$, respectively. Both figures indicate a slight change in the ground state energy, electron probability distribution, and width (depth) of the AHO potential, shifting toward higher energies owing to the $\beta_{2}$ and $\beta_{3}$ effects. However, in the presence of ILF ($\alpha_{0}\neq 0$), Fig. 1c‒f demonstrate that both the width and depth of the HO potential can be changed by varying either the ILF or k, owing to the variation of the degree of AHO harmonicity. Furthermore, as shown in Fig. 1c, the presence of an ILF can transform the AHO potential into an HO potential by reducing its proper depth. It is also noted that both the width and depth of the AHO potential can be decreased by increasing the ILF and the parameters $\beta_{2}$ and $\beta_{3}$, while increasing the parameter *k* leads to improvement (reduction) of the HO width and depth depending on the values of the other parameters (Fig. 1d and 1f). Overall, it is found that the width, depth, and harmonicity of this potential can be driven suitably with an appropriate choice of the above-discussed parameters including the ILF. In the absence of ILF $\left(\alpha_{0}=0\right)$, the changes in the energy difference (electronic transition energy) and its corresponding square dipole matrix element of an AHO potential versus the values of $\beta_{2}$ and $\beta_{3}$ parameters for k=1 are given in Fig. 2a and b, respectively. It is noticed that the transition energy $\left(E_{21}\right)$ increases with the increase of both parameters $\beta_{2}$ and $\beta_{3}$, while this improvement is more marked and faster under the effect of $\beta_{2}$. This is due to the fact that in the absence of ILF, the increase in $\beta_{2}$ has an important impact on the both width and depth of AHO potential, compared to $\beta_{3}\text{.}$ The augmentation in $\beta_{2}$ reduces the well width and also its depth; therefore, the transition energy can only increase within the system. However, it is observed that the square of the dipole matrix element ($M_{21}^{2}$) that gives the oscillation strength between the considered electron states (|1s>→|2p>) show an opposite behavior. The $M_{21}^{2}$ drops in a monotonic way concerning both $\beta_{2}$ and $\beta_{3}$ parameters, while the effect of $\beta_{3}$ is more pronounced than that of $\beta_{2}$. This is because the interaction between the allowed states is greater by reducing the values of $\beta_{3}$ parameter compared to $\beta_{2}$, due to the enhancement in both well width and depth, respectively. In addition, we displayed in Fig. 2b and 2e the same physical parameters ($E_{21},M_{21}^{2}$) of a HO potential as a function of the k parameter in the presence of the ILF. Regarding the ILF, it is obvious that the variation of $E_{21}$ together with k parameter exhibits two behaviors (regimes). It first diminishes rapidly to reach a minimum around k∼1.2, then it begins to increase smoothly, to remain practically constant for k≥3, so $E_{21}$ is increased by about 10meV together with k:∼1.2→3. This occurs because, with the increase of k in this range, the spatial confinement improves, and as a result, the transition energy increases. Moreover, it is observed that this quantity ($E_{21}$) augments quasi-linearly with increasing the ILF intensity, especially for k≤2. It becomes, however, less sensitive to ILF for larger values of *k*, k≥2. When the value of k exceeds 2, the spatial quantum confinement is amplified, leading to an electron wave function that becomes less sensitive to ILF intensity.

**Fig. 3:**
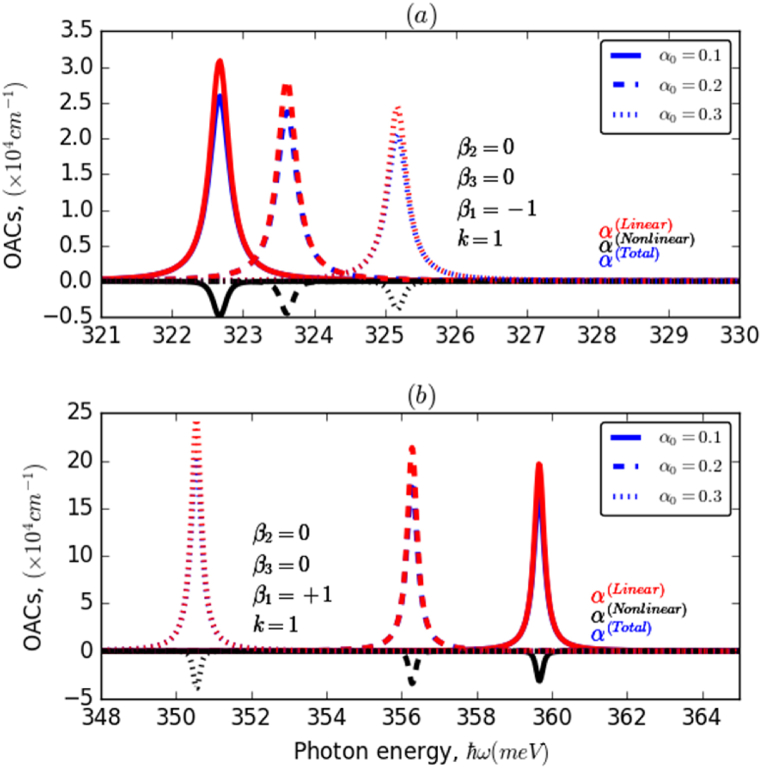
The variation of OACs of the electron confined in the HO potential as a function of the incident photon energy for different values of the ILF intensity $\left(\alpha_{0}=0.1;0.2;0.3\right)$ with k=1 for two cases of $\beta_{1}$: (a) $\beta_{1}=-1$ and (b) $\beta_{1}=+1$.

**Fig. 4:**
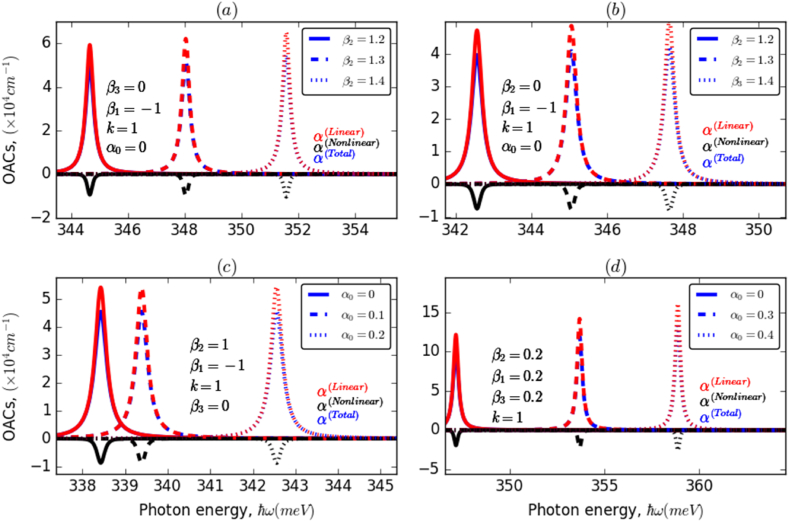
The variation of OACs of an electron confined in AHO potential as a function of the incident photon energy for different values of $\beta_{1},\beta_{2}\text{,}$ and $\beta_{3}$ and ILF ($\alpha_{0}$) parameters, with k=1. (a) and (b) investigate the impact of $\beta_{2}$, while (c) and (d) explore the influence of ILF.

However, it is obvious from the same figures that regardless of the ILF, $M_{21}^{2}$ falls almost exponentially until it reaches a stable value for large values, k≥3, due to the reduction in the overlapping between the involved electronic states as a result of increasing parameter k. Additionally, the incorporation of ILF leads to a decrease in $M_{21}^{2}$; this drop is more marked for values of k smaller than 2 due to the weaker spatial confinement in this region. Furthermore, we plotted the same above-discussed physical quantities ($E_{21},M_{21}^{2}$) of an AHO potential as a function of k parameter and that of the ILF in Fig. 2c and 2f, respectively. It is observed that the transition energy decreases concerning both *k* and ILF strength, respectively. These behaviors are more pronounced for values around 1≤k≤3, which is due to the higher spatial confinement (stronger confinement regime) for values of k in this range, compared to higher values. It is expected that in this region, the more k increases the stronger the confinement will become, and the electron will gain more energy until it will be able to escape from the well to the barrier material. Therefore, it will become more localized within the barrier, and as a result, its energy will decrease.

Thus, it can be seen from the last panel of the same figure (Fig. 1f) that as the parameter *k* increases, the electron wave functions become more localized, which reduces their overlap and in turn, the dipole matrix element. This is because spatial confinement dominates the influence of ILF, leading to a stable value of the dipole matrix element. On the other hand, when *k* is close to 1, the electron wave functions are less confined and display a greater overlap, leading to a minimum in the dipole matrix element. Additionally, when ILF approaches zero, the electron wave functions become less localized and the dipole matrix element increases. Now, to discuss the influence of those parameters on the OACs of both HO and AHO, it is also crucial to mention that $E_{21}$ and $M_{21}^{2}$ are two competitive parameters that govern the behavior of the resonance peaks' position, as well as their amplitudes associated with OACs under various excitations. In addition, according to Eqs. (4−6), the maximum of resonance peaks (OACs related) occurs at an energy $\hbar \omega ={\left[E_{21}^{2}+{\left({\hbar \Gamma }_{21}\right)}^{2}\right]}^{1/2}$, while its associated intensity is proportional to the quantity ${\left[E_{21}^{2}+{\left({\hbar \Gamma }_{21}\right)}^{2}\right]}^{1/2}/{\hbar \Gamma }_{21}$. Under the assumption that $E_{21}\gg {\hbar \Gamma }_{21}$, the energy position of the OACs peak is given by $\hbar \omega \approx E_{21}+{\hbar \Gamma }_{21}$. To deeply understand the effect of the ILF and other considered parameters on the position and/or amplitude of the OACs resonance peaks, we plot in Fig. 3a and 3b the variation of OACs of the electron confined in HO potential as a function of the incident photon energy, for different values of the ILF strength with k=1 and $\beta_{1}=\pm 1$. It is evident that both the amplitude and position of the resonance peaks are affected by the application of ILF. When $\beta_{1}$ has a negative value (3a), the augmentation of the ILF leads to a decrease in the amplitude of the resonance peaks and a blueshift of their positions, while for a negative value of the parameter $\beta_{1}$ (3b), the opposite behavior can be observed concerning ILF.

**Fig. 5:**
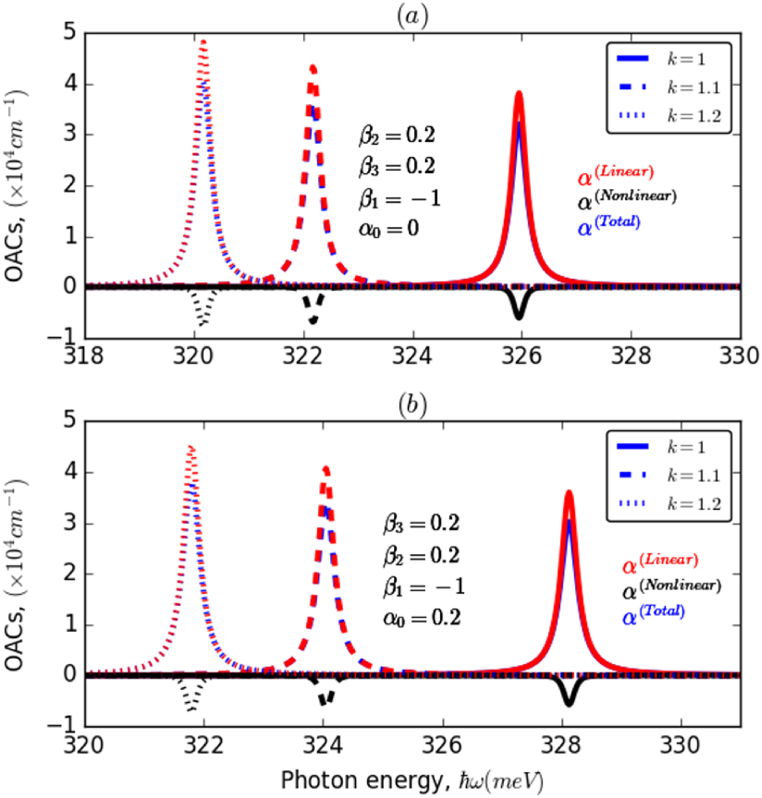
The variation of OACs of an electron confined in AHO potential as a function of the incident photon energy for different values of *k* parameter, without and with ILF. (a) Explores the influence of k in the absence of ILF ($\alpha_{0}=0$), while (b) analyzes the impact of k with ILF ($\alpha_{0}\neq 0$).

This is the result of an increase in the electron transition energy and a decrease in the overlap of states involved as the ILF intensity increases. This behavior can be reversed by changing the value of $\beta_{1}$ from negative to positive. In the first case ($-\beta_{1}$), a blueshift of the resonance peaks of about 3meV was obtained, whereas in the second case ($+\beta_{1}$), a significant redshift of about 10meV was observed. Furthermore, it is noticed that the resonance peaks shifted towards higher energy values by about 40meV and their amplitudes are almost eight times greater owing to the improvement in the overlap between the electronic wave functions. Fig. 4a‒d illustrate the change in OACs-related resonance peaks, associated with an electron confined in AHO potential versus the incident photon energy, taking into account the effects of ILF and $\beta_{2}$ and $\beta_{3}$ parameters for two different values of $\beta_{1}$ with k=1. It can be seen from these panels that the OACs resonance peaks are considerably affected by the applied ILF and considered investigated parameters. Regardless of the ILF intensity (4a,4b), the resonance peaks have been blue-shifted according to values of both $\beta_{2}$ and $\beta_{3}$ with a slight increase in their amplitudes, which is due to the increase in both electron transition energy and strength of the interaction between the electron wave functions of the studied states. This impact is substantially more marked in the case of the β_2_ parameter, compared to β3, which can be explained by the same reason as in the case of $E_{21}$ and $M_{21}^{2}$. Moreover, a similar behavior has been observed in the presence of ILF (4c,4d); it is noticed that this behavior is more pronounced in the case of positive $\beta_{2}\left(4c\right)$ compared to the case of negative $\beta_{1}\left(4d\right)$. In addition, with rinsing the ILF intensity, $\alpha_{0}:0\to 0.3$, the resonance peaks undergo a shift towards higher energy values, of about 4meV in the case of negative $\beta_{1}$, and 13meV in the opposite case. To examine the influence of the k parameter on the OACs resonance peaks, we presented in Fig. 5a and 5b the variation of OACs of an electron confined in AHO potential as a function of the incident photon energy for three different values of k in the absence and presence of ILF, $\alpha_{0}=0,0.2$. As a preliminary observation, it can be seen that changes in the value of the dimensionless k parameter have a marked effect on both the location and height of the resonant peaks of the system's OACs. This influence is slightly lower in the presence of ILF (5b), compared to the case without ILF (5a). Overall, it has been observed that the resonance peaks have shifted to a lower energy range with a significant increase in their amplitudes as the k parameter increased. In the absence of ILF, a redshift of about 6meV is observed. In contrast, the shift is slightly smaller in the presence of ILF. Additionally, the amplitude of OACs is slightly lower in the presence than in the absence of ILF.

**Fig. 6:**
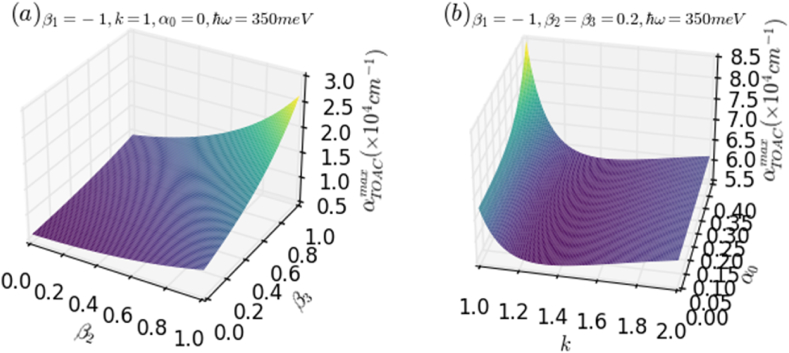
The evolution of TOAC amplitude for the electron confined in the AHO potential as a function of $\beta_{3}\;\left(X-axis\right)$ and $\beta_{2}\;\left(Y-axis\right)$ (a), and as a function of ILF intensity ${[\alpha }_{0}\;\left(X-axis\right)]$ and k(Y−axis) (b), with a fixed photon energy, ℏω=350meV..

To better understand the influence of the ILF and of the key parameters, that determine the harmonicity of the potential, on the amplitude of the total optical absorption coefficient, we have presented two figures (6a and 6b). In Fig. 6a, the variation of the maximum of TOAC for an electron confined in the AHO potential is shown as a function of $\beta_{3}$ on the X-axis and $\beta_{2}$ on the Y-axis. Similarly, in Fig. 6b, the change in the amplitude of TOAC is depicted as a function of $\alpha_{0}$ (ILF intensity) on the X-axis and *k* parameter on the Y-axis, with a fixed photon energy of ℏω=350meV.

**Fig. 7:**
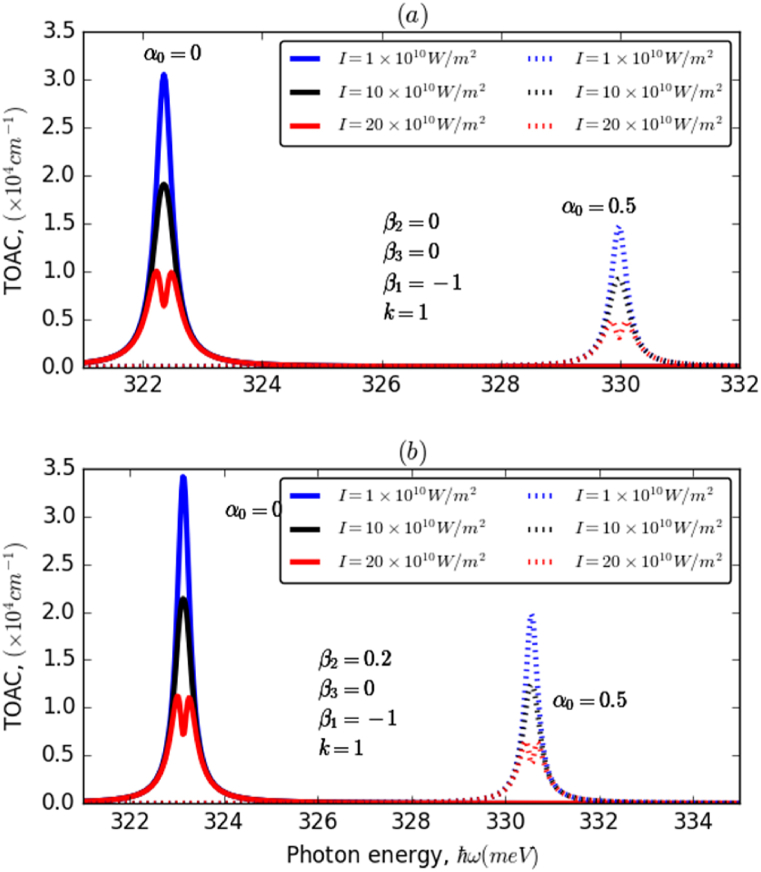
The variation of total OAC of an electron confined in both HO (a) and AHO (b) potentials as a function of the incident photon energy for different values of the incident electromagnetic intensity ($I=1,10,20\;W/m^{2}$) with $\beta_{1}=-1$ and k=1 in the absence of ILF $\left(\alpha_{0}=0\right)$ and in the presence of ILF $\left(\alpha_{0}=0.5\right)$: (a) for $\beta_{2}=0$ while (b) for $\beta_{2}\neq 0$.

It can be inferred from the last figures that all the considered parameters have a remarkable impact on the behavior of the TOAC amplitude. It increases together with increasing both $\beta_{2}$ and $\beta_{3}$, which confirms the results discussed above, and especially, it exhibits a maximum value for $\beta_{2}=\beta_{3}=1\text{.}$ This is a result of the increased anharmonicity of the AHO potential, determined by the increase of the above-mentioned parameters. However, it is important to note that the maximum value of the TOAC decreases as the overlapping between the two considered electronic states reduces with increasing ILF intensity. The behavior of the TOAC is characterized by two regimes concerning the k parameter. This is attributed to the fact that as k increases, both the depth and width of the AHO potential decrease, along with the square dipole matrix element. However, for higher values of the k− parameter, the square dipole matrix element starts to increase again. Fig. 7a and b shows the variation of total OAC of an electron confined in HO and AHO potentials as a function of the incident photon energy for three different values of the incident electromagnetic intensity (*I*), in the presence and absence of the ILF. As can be inferred from these figures, the incident electromagnetic intensity has a significant impact on the TOAC in both cases, with and without ILF, particularly on the maximum of its resonance peak. It is clearly seen that the increase in I reduces the intensity of TOAC and leads to the appearance of two peaks for high electromagnetic intensities, up to $20W/m^{2}$. Furthermore, it has been observed that the effect of the incident electromagnetic intensity is more noticeable in the absence than in the presence of the ILF. It is remarked that by including the ILF ($\alpha_{0}:0\to \;0.5$) in HO potential (7a), the TOAC shifted by about 8meV, from almost 322 to 330meV. However, a slight blueshift of the TOAC-related resonance peak associated with a significant increase in its amplitude has been observed in the case of AHO potential (7b). This is due to the enhancement in the dipole matrix element in the AHO potential compared to the HO one. Our results show a remarkable agreement with previous theoretical studies regarding the effect of ILF on the optical properties in the case of both HO and AHO potentials [30,33,35]. The comparison between GaN/InGaN/GaN and GaAs/AlGaAs semiconductor structures reveals similarities in quantum confinement and tailorability using external excitations such as laser field, but differences in material composition, absorption profiles, and applications. GaN typically has a band gap ranging from approximately 3.4 to 3.6 eV, while InGaN alloys can span a wide range of band gaps depending on the indium composition. For example, InN has a band gap of approximately 0.6–0.7 eV. On the other hand, GaAs have a band gap of around 1.4 eV. The AlGaAs material has a tunable band gap depending on the aluminum composition. The distinct band gap values result in variations in the wavelengths of light absorbed by these materials. GaN-based materials, with a larger band gap, tend to absorb shorter wavelength light (e.g., in the blue or ultraviolet region), while GaAs-based materials, with a smaller band gap, absorb longer wavelength light (e.g., in the infrared region). Understanding these distinct optical properties enables researchers and engineers to optimize device performance and functionality for specific applications, such as LEDs, laser diodes, photodetectors, high-speed electronics, and telecommunications devices. This optimization fosters advancements in communication technology, sensing, and solid-state lighting. Overall, the study's findings provide valuable guidance for designing innovative optoelectronic devices with improved characteristics to meet specific requirements.

## 4 Conclusions

In this study, we investigated the electronic and optical properties of InGaN/GaN nanostructures with harmonic and anharmonic potentials under the effects of an intense laser field (ILF). We used a theoretical approach to analyze the beam quality parameters, including spatial intensity distribution. Our results showed that the electronic and optical properties can be tailored to fit specific responses by adjusting structural parameters and applying laser intensity. We also found that the incident electromagnetic intensity has a more noticeable effect in the absence of the ILF. This causes a decrease in the amplitude of the resonance peaks of the optical absorption coefficients and a displacement towards a higher energy range. Our study provides valuable insights into the electronic and optical properties of the investigated system. This knowledge can be used to design advanced optoelectronic devices with improved performance, such as high-power laser diodes, lasers for nonlinear optics, solar cells, and optical sensors.

## Data availability statement

Data will be made available on request.

## Funding

Not applicable.

## CRediT authorship contribution statement

**Redouane En-nadir:** Writing - review & editing, Writing - original draft, Visualization, Methodology, Investigation, Formal analysis, Data curation, Conceptualization. **Mohamed A. Basyooni-M. Kabatas:** Writing - review & editing, Writing - original draft, Visualization, Validation, Resources, Investigation, Formal analysis. **Mohammed Tihtih:** Writing - review & editing, Visualization, Validation, Methodology. **Haddou El Ghazi:** Writing - review & editing, Validation, Supervision, Software, Resources, Methodology, Investigation.

## Declaration of competing interest

The authors declare that they have no known competing financial interests or personal relationships that could have appeared to influence the work reported in this paper.

## References

[1] Sari H., Kasapoglu E., Sakiroglu S., Yesilgul U., Ungan F., Sökmen I. (2017). Combined effects of the intense laser field, electric and magnetic fields on the optical properties of n-type double δ-doped GaAs quantum well. Phys. E Low-Dimens. Syst. Nanostructures.

[2] Restrepo R.L. (Nov. 2015). Intense laser field effects on a Woods–Saxon potential quantum well. Superlattice. Microst..

[3] Turkoglu A., Aghoutane N., Feddi E., Mora-Ramos M.E., Ungan F. (Aug. 2021). Non-resonant intense laser field effect on the nonlinear optical properties associated to the inter- and intra-band transitions in an anharmonic quantum well submitted to electric and magnetic field. Solid State Commun..

[4] Liu W., Liu Z., Zhao H., Gao J. (Sep. 2023). A simulation study of carrier capture ability of the last inGaN quantum well with different indium content for yellow-light-emitting inGaN/GaN multiple quantum wells. Micromachines.

[5] Sheen M.-H. (Jan. 2023). Correlation between the surface undulation and luminescence characteristics in semi-polar 112‾2 inGaN/GaN multi-quantum wells. Nanomaterials.

[6] Roul B., Chandan G., Mukundan S., Krupanidhi S.B. (2018).

[7] Stonas A.R., Margalith T., DenBaars S.P., Coldren L.A., Hu E.L. (2001). Development of selective lateral photoelectrochemical etching of InGaN/GaN for lift-off applications. Appl. Phys. Lett..

[8] Belmabrouk H. (2020). Modeling the simultaneous effects of thermal and polarization in InGaN/GaN based high electron mobility transistors. Optik.

[9] En-nadir R. (Dec. 2022). Theoretical study of the non-parabolicity and size effects on the diamagnetic susceptibility of donor impurity in Si, HgS and GaAs cylindrical quantum dot and quantum disk: applied magnetic field influence is considered. Philos. Mag. A.

[10] En-nadir R. (Jun. 2023). Exploring the electronic properties of shallow donor impurities in modified ∩-shaped potential: effects of applied electric field, parabolicity, compositions, and thickness. Eur. Phys. J. B.

[11] En-nadir R. (Aug. 2023). Tailoring optoelectronic properties of InGaN-based quantum wells through electric field, indium content, and confinement shape: a theoretical investigation. Phys. B Condens. Matter.

[12] En-nadir R. (May 2023). Analyzing the combined influences of external electric field, impurity-location, in-content, and QW's number on donor-impurity binding energy in multiple quantum wells with finite squared potential. Opt. Quant. Electron..

[13] Abboudi H., El Ghazi H., Benhaddou F., En-Nadir R., Jorio A., Zorkani I. (Feb. 2022). Temperature-related photovoltaic characteristics of (In,Ga)N single-intermediate band quantum well solar cells for different shapes. Phys. B Condens. Matter.

[14] Zscherp M.F. (2023). Overcoming the miscibility gap of GaN/InN in MBE growth of cubic in x Ga1–x N. ACS Appl. Mater. Interfaces.

[15] Tanizaki Y., Koike T. (2014). Real-time Feynman path integral with Picard–Lefschetz theory and its applications to quantum tunneling. Ann. Phys..

[16] Teichmann K., Wenderoth M., Prüser H., Pierz K., Schumacher H.W., Ulbrich R.G. (2013). Harmonic oscillator wave functions of a self-assembled InAs quantum dot measured by scanning tunneling microscopy. Nano Lett..

[17] Wagner E., Shana’a O., Rebeiz G.M. (2019). A very low phase-noise transformer-coupled oscillator and PLL for 5G communications in 0.12$$\backslash$mu $ m SiGe BiCMOS. IEEE Trans. Microw. Theor. Tech..

[18] Labaye F. (2019). XUV sources based on intra-oscillator high harmonic generation with thin-disk lasers: current status and prospects. IEEE J. Sel. Top. Quant. Electron..

[19] Hasanuzzaman G.K.M., Iezekiel S., Kanno A. (2020). W-band optoelectronic oscillator. IEEE Photonics Technol. Lett..

[20] Qi X.-Q., Liu J.-M. (2011). Photonic microwave applications of the dynamics of semiconductor lasers. IEEE J. Sel. Top. Quant. Electron..

[21] Zhang L., Cole J.M., Waddell P.G., Low K.S., Liu X. (2013). Relating electron donor and carboxylic acid anchoring substitution effects in azo dyes to dye-sensitized solar cell performance. ACS Sustain. Chem. Eng..

[25] Malik H.K., Gill R. (Aug. 2017). Terahertz radiation generation in magnetized plasma under relativistic effect. Phys. Plasmas.

[26] Kumar S., Kant N., Thakur V. (Feb. 2023). THz generation by self-focused Gaussian laser beam in the array of anharmonic VA-CNTs. Opt. Quant. Electron..

[27] Kumar S., Thakur V., Kant N. (2023). Magnetically enhanced THz generation by self-focusing laser in VA-MCNTs. Phys. Scr..

[28] Landi G.T., de Oliveira M.J. (May 2013). Fourier's law from a chain of coupled anharmonic oscillators under energy-conserving noise. Phys. Rev. E.

[29] Fletcher N.H. (Dec. 2002). Harmonic? Anharmonic? Inharmonic?. Am. J. Phys..

[30] Mora-Ramos M.E., Duque C.A., Kasapoglu E., Sari H., Sökmen I. (2012). Linear and nonlinear optical properties in a semiconductor quantum well under intense laser radiation: effects of applied electromagnetic fields. J. Lumin..

[31] Ozturk E. (2015). Linear and nonlinear optical absorption coefficients and refractive index changes in double parabolic-square quantum well as dependent on intense laser field. Eur. Phys. J. Plus.

[32] Yesilgul U. (Aug. 2016). Linear and nonlinear optical properties in an asymmetric double quantum well under intense laser field: effects of applied electric and magnetic fields. Opt. Mater..

[33] Panek P.T., Hoeske A.A., Jacob C.R. (Feb. 2019). On the choice of coordinates in anharmonic theoretical vibrational spectroscopy: harmonic vs. anharmonic coupling in vibrational configuration interaction. J. Chem. Phys..

[34] Balsa R., Plo M., Esteve J.G., Pacheco A.F. (Oct. 1983). Simple procedure to compute accurate energy levels of a double-well anharmonic oscillator. Phys. Rev. D.

[35] Chaudhuri R.N., Mondal M. (Sep. 1995). Eigenvalues of anharmonic oscillators and the perturbed Coulomb problem in N-dimensional space. Phys. Rev. A.

[36] Sous A.J. (Jul. 2006). Solution for the eigenenergies of sextic anharmonic oscillator potential v(x)=a6x6+a4x4+a2x2. Mod. Phys. Lett. A.

[37] Adelakun A. (Oct. 2014). Solution of quantum anharmonic oscillator with quartic perturbation. Adv. Phys. Theor. Appl..

[38] Fernández F.M. (Jun. 1992). Strong coupling expansion for anharmonic oscillators and perturbed Coulomb potentials. Phys. Lett..

[39] Patnaik P.K. (May 1986). Perturbation theory for an anharmonic oscillator. Phys. Rev. D.

[40] Ikhdair S.M., Sever R. (Oct. 2007). An alternative simple solution of the sextic anharmonic oscillator and perturbed coulomb problems. Int. J. Mod. Phys. C.

[45] Alaydin B.O., Altun D., Ozturk O., Ozturk E. (Nov. 2023). High harmonic generations triggered by the intense laser field in GaAs/AlxGa1-xAs honeycomb quantum well wires. Mater. Today Phys..

[46] Maleki S., Haghighatzadeh A., Attarzadeh A. (Apr. 2023). Linear and nonlinear optical absorption coefficients and refractive index changes of GaAs/GaAsSb/GaAs V-shaped quantum wells affected by intense laser fields. Opt. Quant. Electron..

[47] Haghighatzadeh A., Attarzadeh A. (Sep. 2023). A comprehensive investigation on valence-band electronic structure and linear and nonlinear optical properties of a laser-driven GaAsSb-based Rosen–Morse quantum well. Eur. Phys. J. B.

[48] Haghighi S., Haghighatzadeh A., Attarzadeh A. (Sep. 2023). Modeling of electronic spectra and optical responses of a laser-affected double GaAsSb/GaAs parabolic quantum well using COMSOL multiphysics: the role of position-dependent effective mass and the static electric field. Opt. Quant. Electron..

[49] Duque C.M., Mora-Ramos M.E., Duque C.A. (Jun. 2013). Properties of the second and third harmonics generation in a quantum disc with inverse square potential. A modeling for nonlinear optical responses of a quantum ring. J. Lumin..

[50] Kasapoglu E. (2015). Combined effects of intense laser field, electric and magnetic fields on the nonlinear optical properties of the step-like quantum well. Mater. Chem. Phys..

[51] Duque C.A., Kasapoglu E., Sakiroglu S., Sari H., Sökmen I. (Oct. 2010). Intense laser effects on donor impurity in a cylindrical single and vertically coupled quantum dots under combined effects of hydrostatic pressure and applied electric field. Appl. Surf. Sci..

[52] Ungan F., Restrepo R.L., Mora-Ramos M.E., Morales A.L., Duque C.A. (Feb. 2014). Intersubband optical absorption coefficients and refractive index changes in a graded quantum well under intense laser field: effects of hydrostatic pressure, temperature and electric field. Phys. B Condens. Matter.

[53] Yücel M.B., Kasapoglu E., Duque C.A. (Jan. 2022). Effects of intense laser field on electronic and optical properties of harmonic and variable degree anharmonic oscillators. Nanomaterials.

[54] Lima F.M.S., Amato M.A., Nunes O.a.C., Fonseca A.L.A., Enders B.G., da S Jr E.F. (Jun. 2009). Unexpected transition from single to double quantum well potential induced by intense laser fields in a semiconductor quantum well. J. Appl. Phys..

[57] Panda M., Das T., Panda B.K. (Feb. 2018). Nonlinear optical properties in the laser-dressed two-level AlxGa1−xN/GaN single quantum well. Int. J. Mod. Phys. B.

[58] El Ghazi H., Jorio A., Zorkani I. (2014). Linear and nonlinear intra-conduction band optical absorption in (In, Ga) N/GaN spherical QD under hydrostatic pressure. Opt Commun..

[59] En-nadir R., Ghazi H.E., Belaid W., Jorio A., Zorkani I., Kiliç H.Ş. (2021). Ground and first five low-lying excited states related optical absorption in In.1Ga.9N/GaN double quantum wells: temperature and coupling impacts. Solid State Commun..

[60] En-nadir R. (Nov. 2022). The electric and magnetic field effects on the optical absorption in double QWs with squared, U-shaped and V-shaped confinement potentials. Philos. Mag. A.

[61] En-nadir R., El-ghazi H. (Jan. 2023). Theoretical study of ISB conduction optical absorption and impurity binding energy associated with lowest excited states in QW with a new modulated potential. J. Theor. Appl. Phys..

[62] En-nadir R. (2022). Intrasubband-related linear and nonlinear optical absorption in single, double and triple QW: the compositions, temperature and QW's number effects. Philos. Mag. A.

[63] Pradhan B., Panda B.K. (Mar. 2014). Effect of intense laser field in GaAs/Al x Ga1−x as quantum well. Adv. Sci. Lett..

